# An attenuated, adult case of AADC deficiency demonstrated by protein characterization

**DOI:** 10.1016/j.ymgmr.2024.101071

**Published:** 2024-03-16

**Authors:** Giovanni Bisello, Christiaan G.J. Saris, Rossella Franchini, Marcel M. Verbeek, Michel A.A.P. Willemsen, Massimiliano Perduca, Mariarita Bertoldi

**Affiliations:** a Department of Neuroscience, Biomedicine and Movement Sciences, Section of Biochemistry, University of Verona, Strada Le Grazie 8, 37134 Verona, Italy; b Department of Neurology, Donders Institute for Brain, Cognition and Behaviour, Radboud University Medical Centre, Geert Grooteplein 10, 6525 GA Nijmegen, the Netherlands; c Translational Metabolic Laboratory, Department of Human Gentics, Radboud University Medical Centre, Geert Grooteplein 10, 6525 GA Nijmegen, the Netherlands; d Department of Pediatric Neurology, Radboud University Medical Centre, Geert Grooteplein 10, 6525 GA Nijmegen, the Netherlands; e Department of Biotechnology, University of Verona, Strada Le Grazie 15, 37134 Verona, Italy

**Keywords:** Aromatic amino acid decarboxylase, AADC deficiency, Pyridoxal 5′-phosphate, Compound heterozygosis, Genotype-phenotype correlation

## Abstract

A case of an adult with borderline AADC deficiency symptoms is presented here. Genetic analysis revealed that the patient carries two AADC variants (NM_000790.3: c.1040G > A and c.679G > C) in compound heterozygosis, resulting in p.Arg347Gln and p.Glu227Gln amino acid alterations. While p.Arg347Gln is a known pathogenic variant, p.Glu227Gln is unknown. Combining clinical features to bioinformatic and molecular characterization of the AADC protein population of the patient (p.Arg347Gln/p.Arg347Gln homodimer, p.Glu227Gln/p.Glu227Gln homodimer, and p.Glu227Gln/p.Arg347Gln heterodimer), we determined that: i) the p.Arg347Gln/p.Arg347Gln homodimer is inactive since the alteration affects a catalytically essential structural element at the active site, ii) the p.Glu227Gln/p.Glu227Gln homodimer is as active as the wild-type AADC since the alteration occurs at the surface and does not change the chemical nature of the amino acid, and iii) the p.Glu227Gln/p.Arg347Gln heterodimer has a catalytic efficiency 75% that of the wild-type since only one of the two active sites is compromised, thus demonstrating a positive complementation. By this approach, the molecular basis for the mild presentation of the disease is provided, and the experience made can also be useful for personalized therapeutic decisions in other mild AADC deficiency patients. Interestingly, in the last few years, many previously undiagnosed or misdiagnosed patients have been identified as mild cases of AADC deficiency, expanding the phenotype of this neurotransmitter disease.

## 1 Introduction

Aromatic amino acid decarboxylase (AADC) deficiency (OMIM #608643) is an autosomal recessive neurotransmitter disease caused by variants in the *DDC* gene (GRCh38.p13; chr7:50,447,733–50,576,163) encoding the AADC enzyme responsible for dopamine and serotonin synthesis from L-Dopa and L-5-hydroxytryptophan, respectively [1]. In the vast majority of cases, the disease onset is in the first months of life, and patients develop serious motor and neurodevelopmental symptoms causing severe disability. The disease is often lethal in the first decade [2]. The therapeutic approach is aimed at ameliorating the symptoms through the administration of pyridoxine (the precursor of pyridoxal 5′-phosphate (PLP), the coenzyme of AADC), monoamine inhibitors and dopamine agonists [2]. Recently, gene therapy surgery into putamen with adeno-associated virus type II carrying the cDNA of AADC (Upstaza®) has been approved by the European Union and the United Kingdom for treatment in severe (non-ambulatory, no head control, and no sitting) patients older than 18 months.

Up to now, about 350 patients have been identified carrying pathogenic variants (pathogenic or likely pathogenic in 90% of the identified genotypes) in either homozygosis or compound heterozygosis [3]. New techniques such as whole exome or next-generation sequencing panels increase the identification of new AADC variants [4,5], whose number jumped up from about 100 to 600 during the last year. No genotype-to-phenotype correlation is known unless the founder variant IVS6 + 4 A > T, that is frequent in patients of South Asian descent [2]. Great effort has been made in recent years to understand the molecular basis of the disease by considering, beyond the genetic variants, the AADC protein population synthesized by patients [[6], [7], [8], [9], [10]]. A step forward is provided by the resolution of the crystal structure of human AADC in its active dimeric native PLP-bound form and in the presence of an L-Dopa analog [11], which aims to understand protein regions responsible for the complete or partial loss-of-function.

Notably, patients with homozygous variants will synthesize the functional dimeric AADC enzyme carrying the same amino acid substitution on both polypeptide chains. The situation for compound heterozygous patients whose AADC protein population is composed of two homodimeric and one heterodimeric AADC species is more complex. A prediction functional score for the heterodimer based on structural analyses has been advanced [3,8,10].

Here, we present the case of an adult with borderline AADC deficiency symptoms whose genotype comprises a known pathogenic variant (p.Arg347Gln) [12] on one allele, and a previously uncharacterized variant (p.Glu227Gln) on the other, adding an additional case of mild AADC deficiency to the literature. Diagnosing mild AADC deficiency may be very challenging since patients may present with a broad spectrum of non-specific signs and symptoms, and may unanticipatedly be identified through (screening) genetic analyses since they do not manifest the characteristic signs of the disease [4,5]. Through a combination of bioinformatics analyses, spectroscopic and kinetic characterization of the recombinant homodimeric (p.Arg347Gln and p.Glu227Gln) and heterodimeric (p.Glu227Gln/p.Arg347Gln) AADC enzymes, we succeeded in contributing to the interpretation of the clinical phenotype displayed by the patient presented here. Overall, an integrated crossed-sectional approach is highly desirable to advance the comprehension of this monogenic disorder.

## 2 Materials and methods

### 2.1 Materials

PLP, pyridoxamine 5′-phosphate (PMP), L-Dopa, dopamine, L-Dopa methylester (DME), hydroxylamine hydrochloride, isopropyl-β-d thiogalactopyranoside (IPTG), phenylmethylsulphonyl fluoride (PMSF), trichloroacetic acid (TCA), and protease inhibitor cocktail (P8849) were purchased from Sigma.

### 2.2 Whole exome sequencing (WES) analysis

The patient gave consent for performing whole exome sequencing. Genomic DNA was extracted from peripheral blood. Exome sequencing was performed as described previously [13] using Illumina HiSeq (BGI-Europa), after enrichment with Agilent SureSelectXT Human All Exon 50 Mb Kit. After read alignment (BWA) and variant calling with GATK (SNV) and CoNIFER (CNVs), annotation was done with in-house developed software. A bioinformatic filter for variants from gene packages intellectual disability (*n* = 1692), mitochondrial diseases (*n* = 467), and metabolic disorders (*n* = 739) were selected and analyzed for predicted pathogenicity (see: https://www.radboudumc.nl/en/patient-care/patient-examinations/exome-sequencing-diagnostics/information-for-referrers/exome-panels).

### 2.3 Biochemical analysis in the cerebrospinal fluid (CSF), urine and plasma

Analyses of neurotransmitter metabolites in CSF and serum were performed as previously published [14].

### 2.4 Site-directed mutagenesis

AADC variants were obtained by mutating the template DNA on the pAADChis vector as previously described [9] using the Quick-Change II kit (Agilent) and the appropriate oligonucleotides for p.Glu227Gln: 5’-CAGGAAGCCCTGCAGAGAGACAAAGCGG-3′ and the related reverse complement and for p.Arg347Gln: 5’-CTTATCACTGACTACCAGCATTGGCAGATA-3′ and the related reverse complement.

All mutations (underlined codon) were confirmed by sequence analysis of the whole ORF.

### 2.5 Recombinant protein production and purification

The recombinant homodimeric and heterodimeric proteins were obtained as described [8]. AADC fractions were concentrated, and the unbound coenzyme was removed by extensive washing with 100 mM potassium phosphate buffer pH 7.4, using Amicon Ultra 15 concentrators (Millipore). All species have been cloned with the His and StrepII tag to allow correct comparisons, since the StrepII tag determines a decrease in activity and slight alterations in other collected parameters, as already published [8]. Protein concentration was determined as follow: His-tagged species using an ε_M_ of 1.42·10^5^ M^−1^ cm^−1^ at 280 nm, StrepII-tagged species using an ε_M_ of 1.50·10^5^ M^−1^ cm^−1^ at 280 nm and heterodimeric His/StrepII-tagged species using an ε_M_ of 1.46·10^5^ M^−1^ cm^−1^ at 280 nm [8]. PLP/AADC ratio was assessed by releasing the PLP in 0.1 M NaOH using ε_M_ of 6600 M^−1^ cm^−1^ at 388 nm [15].

### 2.6 Coenzyme binding affinity measurements

Apo proteins were obtained as previously described [6]. The apparent equilibrium dissociation constant for PLP, K_D(PLP)_, was determined by incubating 100 nM apoAADC in the presence of increasing PLP concentrations (from 0.005 to 20 μM) for 15 h at 25 °C (in the dark) in 0.5 M potassium phosphate buffer pH 6.9. Fluorescence spectra were recorded on a Jasco FP-8500 fluorimeter by exciting the protein samples at 280 nm and the progressive quenching of the intrinsic emission was then fitted to the following equation:

$$Y=Y_{\max}\frac{\left[E\right]t+\left[\mathrm{PLP}\right]t+\mathrm{KD}\left(\mathrm{PLP}\right)-\sqrt{{\left(\left[E\right]t+\left[\mathrm{PLP}\right]t+\mathrm{KD}\left(\mathrm{PLP}\right)\right)}^{2}-4\left[E\right]t\left[\mathrm{PLP}\right]t}}{2\left[E\right]t}$$

where [E]t and [PLP]t represent the total AADC and PLP concentrations, respectively, Y refers to the intrinsic quenching changes at each PLP concentration, and $Y_{\max}\;$refers to the plateau relative to a protein sample with all molecules complexed with the coenzyme. Curves fitting was performed using Prism, 8.4.0 (GraphPad), on three independent experiments. Values obtained are the mean ± standard deviation (SD).

### 2.7 Steady-state kinetic parameters for L-Dopa

The kinetic parameters for the decarboxylation of L-Dopa of AADC variants were determined by HPLC as reported [9]. Each species was pre-incubated with PLP at a concentration 10 times higher than the evaluated K_D(PLP)_. The kinetic parameters were calculated at an appropriate L-Dopa concentration range (0.003–5 mM). The final reaction volume was 225 μL in 100 mM potassium phosphate buffer pH 7.4. The mixtures were then quenched with 25 μL of a 100% TCA solution. Proteins were precipitated in ice and removed by centrifugation. Supernatants were analyzed by HPLC using a Gemini C18 column (150 Å, 4.6 mm, Phenomenex, CA, USA) on a Jasco PU-2080 Plus HPLC system equipped with a UV-1570 detector set at 295 nm. Samples were eluted in 100 mM potassium phosphate, pH 2.35, at a flow rate of 1 mL/min. Standard curves of dopamine peak area were prepared with commercially available dopamine. The determination of the kinetic parameters of each AADC variant was carried out in triplicate and determined by fitting the data to the Michaelis-Menten equation using Prism, 8.4.0, (GraphPad). Data are reported as mean ± SD.

### 2.8 Spectroscopic measurements

All spectral measurements were acquired in 100 mM potassium phosphate, pH 7.4, with 100 μM of exogenous PLP at 25 °C. Circular dichroism (CD) measurements were carried out with a Jasco J-715 spectropolarimeter equipped with a Grant LTC2 refrigerated circulator. Near UV–visible spectra were recorded at a scan speed of 50 nm/min, bandwidth 1 nm, response 4 s, data pitch 1 nm, cell length 1 cm, and at a protein concentration of 0.5 mg/mL. All measurements were repeated in triplicate with each spectrum resulting from the average of 3 accumulations. Thermal denaturation was performed by monitoring the dichroic signal at 222 nm of 0.4 mg/mL protein sample. The instrument was set with a bandwidth of 1 nm, response 4 s, data pitch 0.2 °C, cell length 0.1 cm, and a temperature gradient was applied in the range between 25 and 90 °C with a temperature slope of 1 °C/min.

### 2.9 Bioinformatics analyses, molecular graphics*, in silico* mutagenesis and visualization

Conservation analyses were performed with the Consurf Server (https://consurf.tau.ac.il/) using the human AADC (hAADC) amino acid sequence as input data (id: p20711). A conservation score from 1 (variable residue) to 9 (conserved residue) was attributed to each residue. The thermodynamic effect of the single-point replacements in both the homodimers and heterodimer was then assessed by the FoldX program (http://foldxsuite.Crg.eu/) using the PDB file 8ORA as a template. Data are reported as ΔΔG changes (kcal/mol) of the variant dimeric AADC species with respect to the wild-type (WT) dimer. Values are significant if higher than ±0.5 kcal/mol.

Glu227 to Gln and Arg347 to Gln substitutions were obtained and minimized using the Protein Preparation Wizard [16] in the program BioLuminate within Schrödinger Suite 2021–4 and applying the OPLS4 force field (BioLuminate, Schrodinger, LLC, New York, NY, 2021) and using the PDB file 8ORA as a template. Structural analysis, measurements, and figure drawing were carried out using PyMOL 2.2.3 (The PyMOL Molecular Graphics 50 System, Version 2.0 Schrödinger, LLC).

## 3 Results and discussion

### 3.1 Clinical history of the patient and results of the genetic analysis

The patient was a 55-year-old female presented at the out-patient department because of sudden falls and mood swings. During childhood intellectual disability was identified. As a child, she experienced episodes of spontaneous vertical eye movements when fatigued, perhaps pointing to oculogyric crises. She experienced sudden moments of weakness in both legs, increased in frequency the last year. There were no problems walking the stairs. No clear dysautonomic and endocrine features, gastrointestinal symptoms, or sleep disturbances were present.

She has third degree consanguineous parents (mother from mother and mother from father were sisters), two brothers and one sister. Her 3-years older sister was reported to suffer from a more severe intellectual disability and - in contrast to the patient described here - with inability to walk and talk, but details are missing since there was no contact with her. Both brothers are deceased and also had an intellectual disability. The youngest one had not been able to walk and deceased at 34 years of age. The oldest brother died at the age of 50 during sleep.

On neurological examination she presents mild cognitive impairment and bilateral pyramidal signs (pseudobulbar dysarthria, hypertonia, hyperreflexia with short clonus, adductor reflex showed crossed responses and plantar reflex showed a Babinski response). Walking pattern was cautious with widened base. No evident other neurological abnormalities were found, especially eye movements were normal and no extrapyramidal signs (including dystonia) were present.

After diagnosis (see below), suppletion of pyridoxine 100 mg once daily was started and resulted in more energy and less fatigue symptoms.

Screening genetic analysis identified two compound heterozygote AADC variants:

DDC; Chr7(GRCh37):g.50544323C > T; NM_000790.3:c.1040G > A (p.(Arg347Gln)) heterozygote; DDC; Chr7(GRCh37):g.50595870C > G; NM_000790.3:c.679G > C (p.((Glu227Gln)) heterozygote.

The p.Arg347Gln variant was previously described as pathogenic [3,12,14,17]. The p.Glu227Gln variant is not present in the genome aggregation database (gnomAD).

No other mutations were found, especially no homozygous mutations as suspected based on the consanguinity of the parents and the positive family history.

### 3.2 CSF metabolic profile and plasma AADC activity

Examination of CSF of the patient revealed a metabolic profile suggestive of AADC deficiency (Table 1). This CSF profile is characterized by decreased concentration of dopamine and serotonin degradation products (3-methoxy-4-hydroxyphenylglycol (MHPG) and 5-hydroxyindoleacetic acid (5-HIAA)) and elevated concentrations of the substrates before the metabolic block, namely L-dopa and L-5-hydroxytryptophan. In addition, the degradation product of L-dopa, 3-ortho-methyldopa (3-OMD) is markedly increased. Notably, the CSF homovanillic acid (HVA) value falls within the (lower) reference range. This is, however, not unprecedented in AADC deficiency since some patients have been reported to have normal HVA with increased 3-OMD and L-5-HTP [18]. Alternatively, an external source of HVA might - theoretically - be responsible for the normal concentration of HVA [19]. CSF methyltetrahydrofolate (5-MTHF) is at the limit of the reference range. In plasma, increases of L-Dopa and 3-OMD are coupled to an enzymatic activity 20% lower than the lower limit of the reference range. The AADC deficiency guidelines [2] report that heterozygous carriers present an activity value of about 35–40% of the normal. If we consider the normal as the mean of the reference range (about 53 mU/L), the activity measured in the patient is 28% with respect to the mean. This value seems similar, or almost undistinguishable, to that of heterozygous healthy carriers [2].

**Table 1:** CSF and blood metabolic profile.

	CSF result	Ref. range
L-dopa	58	< 20 nM
3-OMD	396	< 50 nM
HVA	98	87–372 nM
MHPG	14	38–70 nM
L-5-hydroxy-tryptophan	64	<25 nM
5-HIAA	21	58–190 nM
5-MTHF	97	42–98 nM
	**Blood result**	**Ref. range**
L-dopa	86	< 40 nM
3-OMD	1919	< 300 nM
AADC enzyme activity	15	19–86 mU/L

Clinical and metabolic data of this compound heterozygous patient reveal on one side some distinctive features of AADC deficiency, on the other some aspects that are borderline to healthy carriers. We thus tried to interpret the collected data on a molecular basis.

### 3.3 Evolutionary and structural characterization of the AADC variant proteins possessed by the patient reveal no distinctive structural alterations

On the basis of the genotype, the AADC proteins theoretically synthesized by the patient are the two homodimers p.Glu227Gln/p.Glu227Gln and p.Arg347Gln/p.Arg347Gln and the heterodimer p.Glu227Gln/p.Arg347Gln It is already known that the c.1040G > A (p.Arg347Gln) variant is one of the most frequent pathogenic variants in AADC deficiency [3]. The Arg347-to-Gln modification generates an AADC species that retains its structural integrity but is catalytically strongly affected [12] due to the location of the amino acid substitution in the structural element loop3, essential to catalysis [11].

While Arg347 is highly conserved (Consurf score = 9), Glu227 has the lowest score of conservation (Consurf score = 1), revealing its variability. While the p.Arg347Gln/p.Arg347Gln homodimer and the p.Glu227Gln/p.Arg347Gln heterodimer are predicted to be as stable as the WT, by *in silico* computational analysis, the p.Glu227Gln/p.Glu227Gln homodimer seems to be more stable with respect to the WT (Table 2), revealing that this amino acid substitution is predicted not to impact AADC thermodynamic stability.

**Table 2:** FoldX prediction of thermodynamic stability of the AADC variants.

variant	Predicted ΔΔG (kcal/mol)⁎	Effect
p.Glu227Gln	−0.64303	stabilizing
p.Arg347Gln	0.325004	insignificant
p.Glu227Gln/p.Arg347Gln	−0.217915	insignificant

⁎ difference in stability between variant and WT protein species. Error is in the range of 0.5 kcal/mol.

**Fig. 1:**
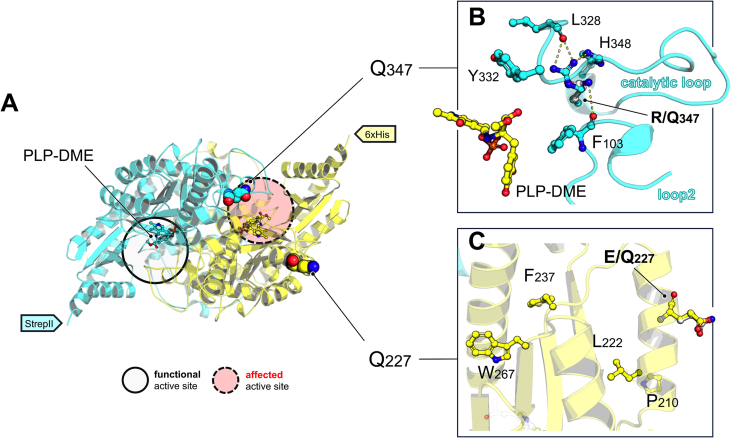
**p.Glu227Gln/p.Arg347Gln AADC heterodimer.** A) AADC dimer (PDB: 8ora). is shown as a cartoon, with one subunit (cyan) harboring residue Gln347 (spheres) and the C-terminal 6xHis tag and the second subunit harboring Gln227 (spheres) and the C-terminal StrepII tail. PLP-DME complex is shown as sticks in both active sites. The predicted functional and affected active sites are circled. B) Arg347Gln was modelled with the AADC structure in the presence of DME (PDB: 8ora). Gln347 (white sticks and cyan spheres) substitutes Arg347 (cyan sticks and blue spheres). C) Glu227 is totally solvent exposed and maps on α-helix on the surface of AADC, distant from the active site, and its side chain is not involved in any interactions (yellow sticks and red spheres). Gln227 (white sticks and red and blue spheres) is not shown to make additional interactions. Amino acid code in the figure is one-letter code.

The structural inspection of the effect of p.Arg347Gln substitution has already been [20] carried out, showing that the variant, present in both [12] or in one of the two [20] subunits, impacts the neighboring active site since it takes place in a structural element (loop3, amino acids 323–357) whose integrity and flexibility is essential for correct catalysis, being the catalytic loop (amino acids 327–341) [11] part of loop3. Arg347-to-Gln substitution perturbs the hydrogen bond network that stabilizes the catalytic loop (Fig. 1A,B). In detail, Gln347 abolishes the contacts that Arg347 stabilizes with Leu328 and His348 concurring to orient the catalytic loop and, in particular, the catalytic residue Tyr332, responsible for the essential reprotonation step in the catalytic mechanism [21]. Furthermore, the substituted Gln347 may generate a new bond with Phe103 backbone, a residue of the nearby structural element loop2 (amino acids 99–109) involved in stacking interactions with the substrate aromatic catechol side chain. For these structural reasons, catalysis is hardly affected.

Glu227 is positioned at the surface of an α-helix belonging to the large domain (composed of amino acids 86–360), totally solvent-exposed and not involved in relevant interactions with other residues. Notably, in close proximity to this surficial helix, there is a hydrophobic β-barrel element where other pathogenic variants, characterized by a low solubility, map [6]: p.Pro210Leu, Leu222Pro, Phe237Ser, and Trp267Arg **(**Fig. 1A,C**)**. However, the loss of solubility is not expected for the p.Glu227Gln substitution, given the surficial position and the retaining of the polar chemical feature. Thus, the p.Glu227Gln/p.Glu227Gln homodimer could be minimally affected by the substitution, while the heterodimer carrying p.Glu227Gln *in trans* with p.Arg347Gln should maintain one active site full functioning, with partial or almost unaffected enzymatic activity.

In a heterodimeric species, one amino acid alteration can positively or negatively complement depending on the amino acid substitution present on the other subunit [10]. A prediction score for heterodimer “fitness” has been suggested considering the number of affected active sites in this enzymatic species [3], thus determining the basis for positive or negative complementation. In order to determine the pathogenicity of the variants present in the genotype, the functional effects of the homodimeric species should be added to that of the heterodimer.

To contribute to unraveling this complex picture, the spectroscopic and functional features of the three species (p.Glu227Gln/p.Glu227Gln homodimer, p.Arg347Gln/p.Arg347Gln homodimer, and p.Glu227Gln/p.Arg347Gln heterodimer) have been determined in order to evaluate the performance of the AADC protein machinery of the patient.

### 3.4 Spectroscopic features and kinetic parameters support a relatively good level of AADC activity in the patient

**Fig. 2:**
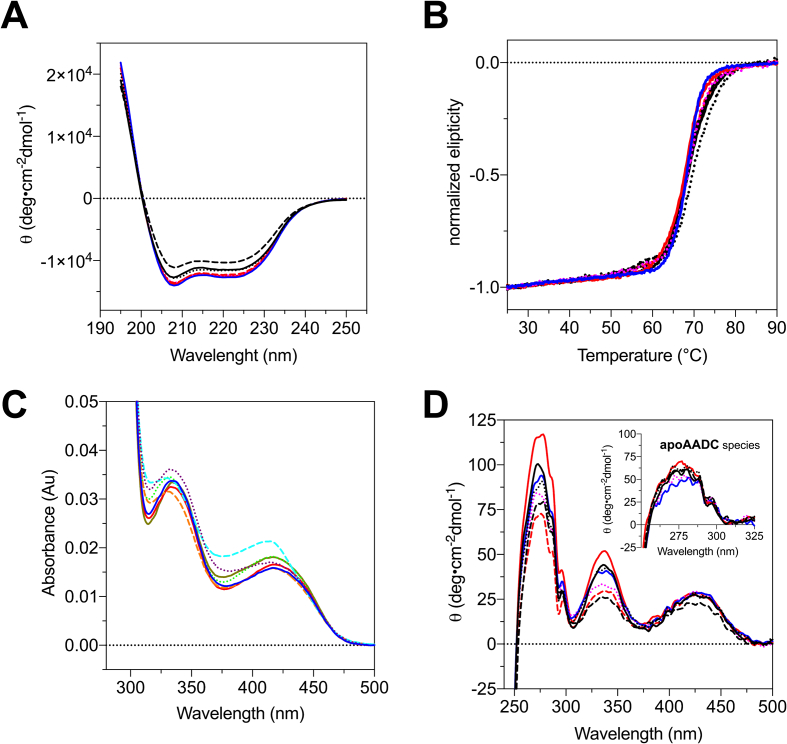
**Spectral features of holo WT and holo AADC variants.** A) Far-UV CD spectra, B) thermal denaturation curves at 222 nm, C) visible absorbance spectra, and D) near-UV visible CD spectra (the inset the near UV spectra of the respective apoenzymes. All experiments were performed in 100 mM potassium phosphate buffer, pH 7.4, at 25 °C, with the addition of 100 μM PLP (excluding the absorbance spectra and the dichroic spectra of the apo species). Each line is the average curve of three independent experiments. Solid lines indicate the homodimeric His-tagged species, the dashed line is the homodimeric StrepII-tagged species, and the dotted line is the heterodimeric His/StrepII-tagged species. Color code: WT species in black, p.Arg347Gln in red, p.Glu227Gln in blue, p.Glu227Gln/p.Arg347Gln in magenta.

The dichroic spectra in the far-UV region show that all enzymatic species retain the same secondary structure features, indicating that the global folding is unaffected by amino acid substitutions or tag binding **(**Fig. 2A**)**. The melting temperature (T_M_) values are an expression of the thermal stability of the protein species and do not show alterations (Fig. 2B **and Table S1**), meaning that all protein species are similarly stable. The absorbance and CD spectra in the visible region (Fig. 2C and D) do not show substantial differences among the purified species, thus suggesting that the PLP microenvironment is minimally affected by amino acid alteration. The near UV CD region witnesses slight alterations in aromatic amino acids orientation dependent on the His-tag rather than the StrepII-tag, as already reported [8], but no significant effects due to the amino acid change. All enzymatic species are able to bind two PLP molecules, and the relative ratios of the spectral signals are similar to those of the WT (**Table S2**). In addition, the equilibrium dissociation constant for the coenzyme is almost unchanged and identical to that of the WT for all species. Altogether, structural features, PLP affinity, and microenvironment are not significantly affected by p.Arg347Gln and p.Glu227Gln substitutions either in homodimeric or heterodimeric arrangements.

The kinetic parameters show that (Table 3) the catalytic efficiency of p.Arg347Gln variant is >10^4^ times lower than that of the WT, mainly for *k*_*cat*_ decrease (about 530 times) than for K_m_ increase (20 times). This is due to a stabilizing effect on catalysis provided by Arg347 that seems to properly orient the catalytic Tyr332 towards the PLP-substrate intermediate [12]. On the contrary, the p.Glu227Gln variant is only faintly affected with a catalytic efficiency comparable to that of the WT species with the same StrepII or His tag. This is expected by the bioinformatic analyses of the structure, given the surficial position of Glu227 and the amino acid alteration to Gln that preserves the chemical polar nature. Interestingly, the p.Glu227Gln/p.Arg347Gln heterodimer presents a catalytic efficiency of 75% with respect to the corresponding His/Strep WT, given by a slight decrease of *k*_*cat*_ and almost intact K_m_ values. Thus, positive complementation takes place in the heterodimeric species because one active site is almost completely preserved.

**Table 3:** Kinetic parameters of decarboxylation of L-Dopa of WT and AADC variants with the different tags.

Species	*k*_*cat*_ (s^−1^)	K_m_ (μM)	*k*_*cat*_/K_m_ (s^−1^ μM^−^1)	K_D_ PLP (nM)
His-WT	5.55 ± 0.23	0.019 ± 0.001	288 ± 16	101 ± 10^a^
StrepII-WT	2.40 ± 0.04	0.028 ± 0.002	86 ± 6	110 ± 20^b^
His/StrepII-WT	3.12 ± 0.20	0.043 ± 0.003	72 ± 4	80 ± 20^b^
His-p.Arg347Gln	0.0105 ± 0.0005	0.401 ± 0.041	0.026 ± 0.002	54 ± 10^c^
His-p.Glu227Gln	5.04 ± 0.16	0.0203 ± 0.0003	249 ± 10	104 ± 6
StrepII- p.Glu227Gln	3.19 ± 0.04	0.034 ± 0.003	93 ± 6	89 ± 8
StrepII-p.Glu227Gln/His-p.Arg347Gln	1.93 ± 0.04	0.037 ± 0.008	53 ± 11	98 ± 8

Data are reported as mean ± SD of three independent experiments.

a Ref [9].

b Ref [8].

c Ref [17].

## 4 Conclusions

Altogether, this compound heterozygous patient presents a picture of a mild form of AADC deficiency with a lack of bilateral extrapyramidal symptoms. The *in silico* and molecular analysis of the purified AADC species shows that the deleterious effects of the pathogenic p.Arg347Gln variant are compensated by the mild p.Glu227Gln substitution, leading to about 67% of the AADC protein population almost properly functioning. This could explain why the plasma AADC activity is only slightly lower than that of heterozygous carriers [2]. The CSF levels of metabolites involved in AADC deficiency present borderline values that are neither typical of a heterozygous carrier nor of a severely affected AADC deficiency patient. To this picture, the lack of bilateral extrapyramidal signs should be added. The amelioration of the fatigue experienced by the patient following pyridoxine treatment probably reflects the general positive effect of vitamin B6 supplementation, also considering that many enzymes involved in amino acids, amines and glycogen metabolism rely on the derivative coenzyme PLP for their proper function [22,23]. The combination of clinical and molecular results provides us with the basis to understand the effects of pharmacological treatment for the AADC variants carried by this patient and could be useful in light of the precision medicine necessary for rare diseases caused by individual alterations. Since gene therapy for mild AADC deficiency patients is not recommended up to now, efforts to set up personalized treatments are necessary.

Notably, caution should be paid to patients presenting borderline AADC deficiency symptoms, alterations in CSF metabolite levels, and also modest decreases in plasma AADC activity. In the last few years, given the availability of next-generation sequencing methods and whole exome or genome analyses, many mild cases have been reported [4,5], suggesting that they could represent the tip of the iceberg since many of them could still be undiagnosed or misdiagnosed.

## Authors contributions

GB performed kinetic, spectroscopic and bioinformatic experiments; data analysis and interpretation; CGJS performed genetic diagnosis and clinical care and assessment of the patient and manuscript drafting; MMV performed biochemical analyses in CSF and plasma enzyme activity, and manuscript drafting; MAAPW performed clinical care and assessment of the patient, and manuscript drafting; RF performed kinetic experiments; MP performed biostructural and computational experiments; MB performed study conceptualization, data interpretation, and manuscript writing.

## Acknowledgments and funding

The technical support of Silvia Bianconi (University of Verona) is gratefully acknowledged. CGJS is a member of the Radboudumc Center of Expertise for Neuromuscular Disorders (Radboud-NMD), the Netherlands Neuromuscular Center (NL-NMD), and the European Reference Network for Rare Neuromuscular Diseases (EURO-NMD). This work has been funded by 10.13039/100013223PTC Therapeutics IIS Grant, by PRIN2022 code: 2022BTMTP8, Ministry of University and Research, Italy, and by FUR2022 10.13039/501100007052University of Verona to MB. MMV is supported by a grant from the Stichting Alkemade-Keuls.

## CRediT authorship contribution statement

**Giovanni Bisello:** Investigation, Formal analysis, Data curation. **Christiaan G.J. Saris:** Writing – original draft, Investigation, Data curation. **Rossella Franchini:** Investigation. **Marcel M. Verbeek:** Writing – review & editing, Supervision, Investigation. **Michel A.A.P. Willemsen:** Writing – review & editing, Supervision, Data curation. **Massimiliano Perduca:** Investigation. **Mariarita Bertoldi:** Writing – review & editing, Writing – original draft, Funding acquisition, Data curation, Conceptualization.

## Declaration of competing interest

M.B. received research funding and unconditional support by PTC Therapeutics, Inc.

## Data Availability

Data will be made available on request.

## Appendix A Supplementary data

Supplementary material Image 1
