# Supplementary material for: An attenuated, adult case of AADC deficiency demonstrated by protein characterization
Source: Mol Genet Metab Rep. 2024 Mar 16;39:101071. doi: 10.1016/j.ymgmr.2024.101071 (PMC10958467; doi:10.1016/j.ymgmr.2024.101071)
Supplement: Supplementary file 1 — Supplementary material [file mmc1.docx]

**Table S1. T_M_ values for the AADC WT and variant species**

| **Species** | **^a^T_m_ θ_222nm_ (°C)** |
| --- | --- |
| His-WT | 68.80 ± 0.14 |
| StrepII-WT | 68.49 ± 0.49 |
| His/StrepII-WT | 70.61 ± 0.15 |
| His-p.R347Q | 68.39 ± 0.31 |
| His-p.E227Q | 67.85 ± 0.29 |
| StrepII-p.E227Q | 68.35 ± 0.12 |
| His-p.E227Q/StrepII-p.R347Q | 68.77 ± 0.08 |

^a^Mean of three independent experiments ± SD

**Table S2. PLP content and microenvironment features evaluated by the ratio 335 nm/420 nm in absorbance and visible CD spectra.**

| Species | PLP/enzyme | Abs_335/420_ | θ_335/420_ |
| --- | --- | --- | --- |
| His-WT | 1.9 | 2.1 | 1.6 |
| StrepII-WT | 1.7 | 2.1 | 1.2 |
| His/StrepII-WT | 1.8 | 1.6 | 1.5 |
| His-p.R347Q | 1.9 | 2.0 | 1.4 |
| His-p.E227Q | 1.9 | 1.9 | 1.8 |
| StrepII-p.E227Q | 1.8 | 2.0 | 1.1 |
| StrepII-p.E227Q/His-p.R347Q | 1.9 | 1.9 | 1.2 |

Data are calculated from the respective spectra and represent mean values of three spectra.

Errors are less than 10% in all cases.
